# Sri Lanka - Health in the middle of a crisis

**DOI:** 10.1016/j.lansea.2022.100067

**Published:** 2022-09-02

**Authors:** The Lancet Regional Health – Southeast Asia

Sri Lanka—once a role model for health and development—is currently in an economic crisis. Attempts at swift development by borrowing enormous amounts of money at high interest rates, banning the import of chemical pesticides to curb outflow of foreign currency, tax cuts, and the effects of the COVID-19 pandemic and the Russia–Ukraine war on tourism have catapulted Sri Lanka into an economic downfall. The economic mismanagement and its consequences are unforgiveable because it has forced millions of people into poverty.

Most importantly, the economic crisis has put national health care in jeopardy. Physicians and health-care workers are trying to keep the Sri Lankan health-care system afloat. However, the current crisis has led to a shortage of fuel affecting the supply of medical equipment and emergency transport of patients and health-care workers. Out of options, nurses are working double shifts because daily transport to work has become unaffordable. With stocks of essential medication reaching a record low, physicians are focusing on ways to procure crucial medication. Sri Lanka used to import most of its medication, but now with a shortage of foreign currency, the hopes of procuring more medication are low. During April, 2022, there was a 40% increase in the cost of medication. Adding to the woe, medication has become up to four times more expensive and unaffordable to many people. Although most hospitals in Sri Lanka are public, the increase in cost of medication would discourage people from using private hospitals and increase burden on the public sector, which might encourage health-care workers to emigrate from the country to seek better jobs.

Shortage of fuel has also increased the food prices and affected nutrition. According to the Demographic and Health Survey 2016–17, 20·5% of Sri Lankan children were underweight in 2016 and there was no substantial improvement from 2006. Unfortunately, reports from the World Food Programme (WFP) suggests that currently 86% of families in Sri Lanka are buying less nutritious and cheaper food. The practice of consuming less food and forgoing meals has also emerged, and if continued, more children, pregnant, lactating women, and older citizens will face malnutrition. Previously, the economic crisis in the former Soviet Union and Zimbabwe resulted in widespread malnutrition and was strongly associated with the increase of tuberculosis cases. If Sri Lanka does not receive help, there is a high chance of such a surge occurring. Availability of clean and potable water is also bound to be an issue. Wastewater treatment would effectively prevent an outbreak of water-communicable diseases, such as cholera. The authorities need to ensure water availability and wastewater facilities to all people without discrimination by religion, region of residence, or political ideology to prevent similar events that occurred in Zimbabwe during the economic crisis in 2009. The COVID-19 pandemic had already led to underreporting of dengue cases in Sri Lanka, and with the absence of funding, epidemiological surveillance of infectious diseases will be a challenge.

Without a doubt, the economic crisis majorly affected the mental health of Sri Lankan people. Schools have closed and regular power disruptions have made online learning difficult. According to the Save the Children Fund in Sri Lanka, closure of schools has led to change in behaviours in children, such as changes in appetite and emotional regulation, an increase in aggressive behaviour, and disturbed sleep patterns. Also, the reduced education attainment would also impact future employment and the nation's economy in the long term. Similarly to the economic collapse of Zimbabwe, reduced income might increase transactional sex in exchange for basic groceries, as emerging reports have already indicated that some women in Sri Lanka are reluctantly becoming sex workers to survive, which could lead to numerous issues, such as an increase in sexually transmitted diseases and more people seeking medical care for mental health-related problems.

Other regional countries, such as Pakistan, are claiming to have averted a major economic crisis, but Bangladesh is still facing economic challenges. To manage debt, India has provided US$3·8 billion to Sri Lanka as financial assistance. The Norwegian government has also allocated 13 million Norwegian krone (around US$1·3 million) to help Sri Lanka's food crisis. However, according to the WFP, Sri Lanka needs a total of US$63 million in the upcoming months for all life-saving activities. Further foreign aid is needed from other countries, regionally and globally, to help Sri Lanka immediately overcome this crisis.

